# The Loss of HLA-F/KIR3DS1 Ligation Is Mediated by Hemoglobin Peptides

**DOI:** 10.3390/ijms21218012

**Published:** 2020-10-28

**Authors:** Gia-Gia T. Hò, Wiebke Hiemisch, Andreas Pich, Georg M. N. Behrens, Rainer Blasczyk, Christina Bade-Doeding

**Affiliations:** 1 Institute for Transfusion Medicine, Hannover Medical School, Carl-Neuberg-Str. 1, 30625 Hannover, Germany; Ho.Gia-Gia@mh-hannover.de (G.-G.T.H.); Hiemisch.Wiebke@mh-hannover.de (W.H.); Blasczyk.Rainer@mh-hannover.de (R.B.); 2 Institute of Toxicology, Hannover Medical School, Carl-Neuberg-Str. 1, 30625 Hannover, Germany; Pich.Andreas@mh-hannover.de; 3 Department of Rheumatology and Immunology, Hannover Medical School, Carl-Neuberg-Str. 1, 30625 Hannover, Germany; Behrens.Georg@mh-hannover.de; 4 German Center for Infections Research, partner site Hannover-Braunschweig, Inhoffenstraße 7, 38124 Braunschweig, Germany

**Keywords:** HLA-F, peptides, proteome, KIR3DS1

## Abstract

The human leukocyte antigen (HLA)-Ib molecule, HLA-F, is known as a CD4^+^ T-cell protein and mediator of HIV progression. While HLA-Ia molecules do not have the chance to select and present viral peptides for immune recognition due to protein downregulation, HLA-F is upregulated. Post HIV infection, HLA-F loses the affinity to its activating receptor KIR3DS1 on NK cells leading to progression of the HIV infection. Several studies aimed to solve the question of the biophysical interface between HLA ligands and their cognate receptors. It became clear that even an invariant HLA molecule can be structurally modified by the variability of the bound peptide. We recently discovered the ability of HLA-F to select and present peptides and the HLA-F allele-specific peptide selection from the proteomic content using soluble HLA (sHLA) technology and a sophisticated MS method. We established recombinant K562 cells that express membrane-bound HLA-F*01:01, 01:03 or 01:04 complexes. While a recombinant soluble form of KIR3DS1 did not bind to the peptide-HLA-F complexes, acid elution of the peptides resulted in the presentation of HLA-F open conformers, and the binding of the soluble KIR3DS1 receptor increased. We used CD4^+^/HIV^−^ and CD4^+^/HIV^+^ cells and performed an MS proteome analysis. We could detect hemoglobin as significantly upregulated in CD4^+^ T-cells post HIV infection. The expression of cellular hemoglobin in nonerythroid cells has been described, yet HLA-Ib presentation of hemoglobin-derived peptides is novel. Peptide sequence analysis from HLA-F allelic variants featured hemoglobin peptides as dominant and shared. The reciprocal experiment of binding hemoglobin peptide fractions to the HLA-F open conformers resulted in significantly diminished receptor recognition. These results underpin the molecular involvement of HLA-F and its designated peptide ligand in HIV immune escape.

## 1. Introduction

The human leukocyte antigen F (HLA-F) is one of the non-classical HLA-Ib molecules (HLA-F, HLA-E and HLA-G). The expression pattern of HLA-F is highly restricted and differs significantly from the classical HLA Ia (HLA-Ia) molecules. Surface expression of HLA-F has only been detected on B cells, T cells and NK cells [1]. HLA-F has been known to be expressed as an open conformer (OC) without peptide and association with β2-microglobulin (β2m) [2,3]. HLA-F OC constitutes the ligand for NK cell receptors such as killer immunoglobulin-like receptor (KIR) 3DS1 (KIR3DS1), KIR3DL1 and KIR3DL2 [4,5]. It has been assumed that HLA-F is entirely unable to present peptides [3], implying HLA-F to display an invariant surface for its cognate receptor. Recently, it has been demonstrated that HLA-F is able to assemble with β2m and to bind and present peptides like classical HLA-I molecules [6,7]. Of note, peptides selected and presented by HLA-F exhibit unusual length [6,8]; further analysis revealed that the peptides are exclusively C terminal anchored; the unique structure of the HLA-F peptide-binding region (PBR) impedes the classical pocket A and pocket B engagement and enables the binding of these long peptides [6,8]. However, peptide-HLA-F complexes (pHLA-F) are ligands for immunoglobulin-like transcript receptor-2 (ILT-2) [6,9]. Thus, HLA-F has the capability to impair NK cell reaction in both an inhibitory and activating way.

Classical HLA-Ia molecules (HLA-A, HLA-B and HLA-C) are key regulators of the immune system and maintain immune surveillance. HLA-Ia molecules are expressed at almost all nucleated cells and monitor the health status of the cell. During pathogenic episodes like viral or bacterial infections, they activate the immune system through the presentation of foreign peptides. Antigen presentation plays an essential role in immune recognition of infected or malignant cells. It becomes obvious that many viruses like human cytomegalovirus (HCMV) and human immunodeficiency virus (HIV) evolved several strategies to avoid recognition by the host immune system, including downregulation of HLA-Ia molecules [10,11]. Downregulation of HLA molecules reduces the inhibitory signals to NK cells resulting in activation of NK cells by missing-self recognition [12]. To evade this immune activation, HCMV-infected cells present the UL40 peptide on HLA-Ib molecule HLA-E. UL40/pHLA-E is a ligand for the inhibitory receptor NKG2A/CD94 on NK cells [13]. Peptides of other viruses also stabilized the HLA-E expression on infected cells to avoid NK cell-mediated toxicity [14,15].

HLA-Ib molecules are marginal polymorphic and are able to mediate immune tolerance instead of immune activation [16,17,18]. During a pathogenic episode where the infected cells want to remain unrecognized, a fine-tuning between downregulation of HLA-Ia and upregulation of HLA-Ib molecules can be observed. To avoid recognition by cytotoxic T cells (CTLs), HIV selectively downregulates HLA-A and HLA-B, while HLA-C and HLA-E remain on the cell surface to evade NK cell-mediated lysis [19]. The role of HLA-F during HIV infection is still an enigma. KIR3DS1 is an HLA class I binding receptor expressed on NK cells [20]. The activating KIR3DS1 NK cell receptor stimulates the secretion of cytokines and IFN-gamma production. It was the first KIR that has been associated with the outcome of viral infection [20]. The interaction between HLA-F and KIR3DS1 leads to the secretion of antiviral cytokines and a slower progression of HIV [5]. However, during the course of HIV infection, the amount of HLA-F increases, while its interaction with KIR3DS1 somehow diminishes [5,21]. These observations lead to the suggestion that HLA-F is involved in immune evading mechanisms. Since only HLA-F OC is a ligand for KIR3DS1 it remains questionable if maybe peptide presentation is involved in this unknown immune evading mechanism. Especially for the restricted HLA-Ib, peptides dictate the direction of immune reaction [22,23,24]. For instance, the potential of HLA-E to present peptides of noncanonical length leads to differential NK cell recognition [24]. Peptide binding stabilizes and determines the structure of an HLA-molecule and, therefore, the accessible recognition surface for immune effector cells [25,26]. For HLA-I molecules, most of the peptides are generated by proteasomal cleavage [27]; consequently, the nature of bound peptide is determined by the available proteome. Yet, it is known that HLA-F can present peptides, but the function of pH LA-F remains an unsolved puzzle. The key role in HLA-Ib immune regulation is mediated by the selection and presentation of peptides. This study aims to provide further knowledge about HLA-F and the role of peptide presentation in this non-classical HLA-I molecule.

## 2. Results

### 2.1. Hemoglobin Subunits were Upregulated in CD4^+^/HIV^+^ T Cells

**Figure 1:**
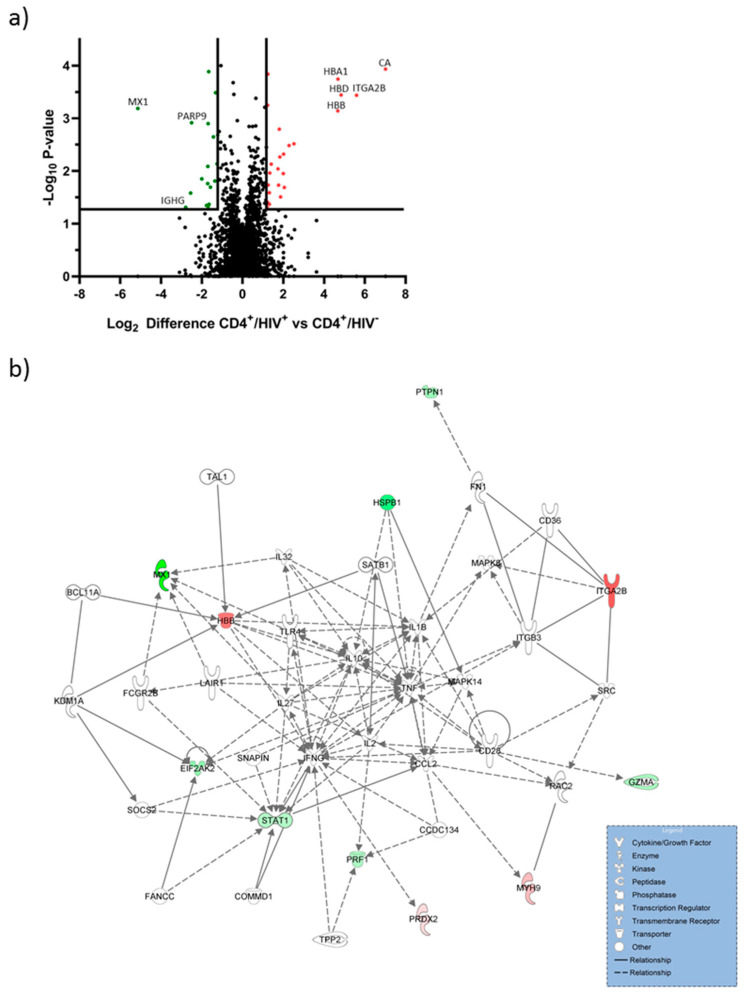
Mass spectrometric analysis of the proteome of CD4^+^ T cells from therapy-naïve patients with HIV and CD4^+^/HIV^−^ T cells. (**a**) Protein abundance in CD4^+^ T cells from therapy naïve-patients infected with HIV. Results are shown as a volcano plot. Protein abundance of three replicates is plotted as log2 value against the negative decadic logarithm of the *p* values. Proteins were regarded as regulated from factor 1.2 and *p* value < 0.05. Significantly upregulated proteins due to HIV infection are shown in red and downregulated proteins in green; (**b**) network analysis for up-and downregulated proteins are illustrated in green, not colored proteins were added by the IPA algorithm. High-confidence interactions are symbolized by a continuous line; medium-confidence interactions are symbolized by a dashed line.

Proteome analyses were performed to analyze the abundance of proteins post HIV infection. The proteome of CD4^+^/HIV^+^ T cells was compared with CD4^+^/HIV^−^ T cells. MS-based proteomic analysis revealed 2963 protein groups, of which 1601 could be quantified. Proteins significantly (*p* value < 0.05) regulated and at least altered by factor log2 ± 1.2 were regarded as regulated (Figure 1a).

A total of 42 of 1601 proteins were significantly upregulated in post-HIV-infected CD4^+^ T cells. The 10 strongest upregulated proteins are shown in Table 1. The 10 strongest downregulated proteins are shown in Table 2. Carbonic anhydrase (CA) that is involved in pathological processes was the strongest upregulated protein [28]. Hemoglobin alpha, beta and delta were significantly upregulated in CD4^+^/HIV^+^ cells. These molecules are involved in O_2_ homeostasis and oxidative stress regulation [29].

The most downregulated protein was interferon-induced GTP-binding protein Mx1 (MX1). MX1 exhibits antiviral properties and plays an important role against a wide range of RNA viruses [30,31]. Protein mono-ADP-ribosyltransferase (PARP9) was also downregulated and is involved in interferon-mediated antiviral defenses [32].

### 2.2. Presentation of HBB Peptide Reduced the Recognition of HLA-F by KIR3DS1

**Figure 2:**
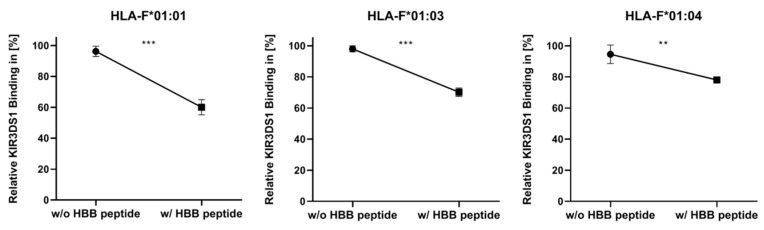
Hemoglobin subunit beta (HBB) peptide binding resulted in diminished KIR3DS1 recognition. K562/F*01:01, K562/F*01:03 *and* K562/F*01:04 cells were analyzed for KIR3DS1 binding with and without HBB peptide. Experiments were performed three times (*n* = 3). Significance of (**) describes a *p* value of *p* < 0.01 and significance of (***) describes a *p* value of *p* < 0.001.

Based on proteome analysis, we could identify hemoglobin subunit beta (HBB) as upregulated during HIV infection. Furthermore, previous peptide sequence analysis from HLA-F allelic variants featured HBB peptides (VNVDEVGGEALGR) as dominant and shared. To investigate the impact of peptide presentation on KIR3DS1, we performed the reciprocal experiment of binding hemoglobin peptide (VNVDEVGGEALGR) to HLA-F and analyzed the binding of recombinant KIR3DS1 (Figure 2).

KIR3DS1 binding was reduced in all three allelic variants following peptide loading. By comparing the three allelic variants, the binding of KIR3DS1 illustrated variability depending on the HLA-F allelic variant. Among HLA-F allelic variants, the KIR3DS1/HLA-F*01:01 engagement showed the strongest decrease following the presentation of an HBB peptide, with 36.22%. In K562/F*01:03, the binding affinity of KIR3DS1 decreased by 27.89% following peptide binding. The least change when using the HBB peptide-loading assay was detected in K562/F*01:04 cells, with 16.47%.

KIR3DS1/HLA-F engagement diminished with peptide binding. Amino acid exchange in heavy chain changed the KIR3DS1/HLA-F/HBB peptide interaction.

## 3. Discussion

The function of HLA-Ia molecules is to scan the intracellular proteome and present peptides to immune effector cells to maintain immune surveillance. During the course of infection, viral immune evasion proteins downregulate HLA-Ia expression [10,11]. Consequently, infected cells do not present pH LA-Ia complexes on the surface and would be susceptible to NK cell-mediated lysis [12]. Non-classical HLA-Ib molecules are upregulated during these infectious periods and protect HLA-Ia empty cells from being recognized by NK cells [33]. Distinct pHLA-E complexes are stabilized on the cell surface and provide a ligand for inhibitory NKG2A/CD94 receptor. The HLA-E:NKG2A/CD94 engagement is peptide-specific; therefore, peptide selection of HLA-E is crucial for pathogenic immune escape mechanisms [23,34]. The biological function and implementation of HLA-G are highly dependent on its structure and bound peptides. Hence, pathogens are using HLA-E and HLA-G molecules for immune escape. In these invariable molecules, peptide specificity determines the reactivity of pHLA complexes with their cognate immune receptors [22,34]. Among non-classical HLA-Ib molecules, HLA-F is unique. HLA-F is invariant, and until recently, it was thought that HLA-F only exists as an open conformer incapable of presenting peptides [5]. HLA-F serves as a ligand for KIR3DS1; the interaction leads to the secretion of antiviral cytokines and a delayed disease progression of HIV. HIV infection causes the phenomenon of HLA-F upregulation [5,21], while other HLA-Ia molecules are downregulated [11]. Furthermore, the interaction between HLA-F and KIR3DS1 diminished [5]. Since the structure of marginal polymorphic HLA-F is relatively invariant and it could be demonstrated that HLA-F is able to present peptides [6,7], peptide presentation may be the key to shed light on the molecular mechanism of this phenomenon. Peptides not only determine the half-life time of the whole pHLA-complex but also dictate specificity and reactivity between HLA molecules and their immune receptors.

Most of the peptides presented by HLA class I molecules are generated by proteasome within the cell. The nature of bound peptides is determined by the available proteome [27]. Thus, the identification of host protein modulation in HIV-infected cells is the first step towards understanding the available peptide repertoire. The proteome coverage of CD4^+^ T cells derived from HIV-infected patients with 1602 quantified protein groups was comparable to other proteome analysis of CD4^+^ T cells [35,36]. HIV infection changed the proteome of CD4^+^ T cells; 20 proteins were significantly upregulated in CD4^+^/HIV^+^ cells. It becomes obvious that the modification of the proteome will also affect the quantity of potential HLA ligands. We could detect hemoglobin as significantly upregulated in CD4^+^ T-cells post HIV infection. Hemoglobin is the major oxygen-transport protein in red blood cells [37]. Apart from red blood cells, cellular hemoglobin is also expressed in nonerythroid cells such as CD4^+^ T cells, neurons and epithelial cells [29,38]. In these cells, hemoglobin plays an important role in the maintenance of O_2_ homeostasis and protection against oxidative stress [29,38]. Proteomic analysis of hemoglobin in erythrocytes demonstrated the interaction of hemoglobin with several proteins such as chaperones, oxidoreductases and metabolic enzymes [39]. Our protein network analysis shows a connection of hemoglobin to IFN-γ and TNF. Hemoglobin-derived peptide has been described as a novel type of bioactive signaling molecules [40]. The presentation of hemoglobin peptides on HLA-Ib molecules has not been described yet. In all investigations concerning the interaction between HLA-F and its cognate immune receptors, only HLA-F*01:01 and HLA-F*01:01 OC were examined [5,6]. Our peptide sequence analysis from HLA-F allelic variants featured hemoglobin subunit beta (HBB) peptide (VNVDEVGGEALGR) as dominant and shared [8]. Since HBB is upregulated during HIV infection and presented by all three allelic variants of HLA-F, we suggest that peptide presentation is involved in the obstacle of reducing interaction between HLA-F and KIR3DS1 in HIV infection. Recently, it could be verified that the proteomic source is significant for peptide selection in invariant HLA-G molecules [17].

In the present study, we investigate KIR3DS1 ligation to HLA-F*01:01, HLA-F*01:03 and HLA-F*01:04 in dependence of HBB peptide binding. HLA-F*01:01, HLA-F*01:03 and HLA*01:04 are distinguished by polymorphism outside the peptide-binding region (p.50P>Q, F*01:04 and p.251S>P, F*01:03) and do not directly interact with peptide binding [41] or binding of known receptors [42]. However, polymorphism of other HLA-Ib molecules HLA-E and HLA-G that should also not interfere with peptide selection, binding, and presentation impacts their immune function significantly [22,34]. Recently, it has been shown that the polymorphism of HLA-F allelic variants does not influence peptide features of presented peptides [8]. The pHLA structure of HLA-F differs from the classical pHLA structure [6,7]. The binding of long peptides that protrude out at the N terminal side of the peptide-binding region leads to the formation of a very flexible, accessible surface of HLA-F. For HLA-E, it could be demonstrated that peptides dictate the interaction partner; depending on the presented peptide, HLA-E constitutes a ligand for inhibitory NKG2A/CD94 receptor or activating NKG2C/CD94 receptor on NK cells [34]. Thus, it becomes obvious that flexible structures of peptide bound HLA-F molecules influence the recognition of its corresponding immune receptor. In this study, we could show that HBB peptide presentation of HLA-F resulted in a significantly decrease of KIR3DS1 binding. Furthermore, we could demonstrate different peptide-binding properties between the three allelic variants. HLA-F*01:01 presenting HBB peptide resulted in the highest reduction of KIR3DS1 engagement. A reason for this observation may be that potential immune evasion strategies of HIV are best evolved in the most common allelic HLA-F variant, HLA-F*01:01. Interestingly, HLA-F*01:04 showed the lowest reduction of KIR3DS1 binding, suspecting its role as more protective HLA-F allelic variant during HIV infection. To confirm this assumption further analysis such as typing of HLA-F in HIV-infected patient would be necessary. Studies about HLA-G could demonstrate that less frequent allelic variant showed significant immune regulation properties; HLA-G*01:04 showed an increased protective potential and a stronger affinity to inhibitory NK cell receptor than HLA-G*01:01 and HLA-G*01:03 [22].

The data presented in this study emphasize the role of protein regulation and peptide presentation for the reactivity of HLA-Ib molecules during pathogenic episodes. Each pHLA-I molecule displays a unique landscape to its cognate immune receptor. These results underpin the molecular involvement of HLA-F and its designated peptide ligand in HIV immune escape. Knowledge about peptide origin and peptide presentation in HLA-F will contribute to a better understanding of HLA-F and its unexplored function in immunity.

## 4. Materials and Methods

### 4.1. Proteome Analysis of CD4^+^ T Cells of HIV-Infected Person

For proteome analysis, 1 × 10^6^ CD4^+^T cells from therapy-naïve patients with HIV (approved by the ethics committee of Hannover Medical School; code 2411–2014) and CD4^+^/HIV^−^ T cells were lysed in 100 µL RIPA buffer as previously described [8]. Protein concentration was ascertained using the bicinchoninic acid assay (BCA) protein quantitation kit (Interchim, San Diego, CA, USA). A total of 50 µg of protein was heated at 95 °C for 5 min, alkylated by adding 1 µL 40% acrylamide at RT and separated by SDS gel electrophoresis. Gels were stained with Coomassi SimplyBlue™ SafeStain (Thermo Fischer Scientific, Waltham, MA, USA). To reduce complexity and thereby increase the amount of identified proteins, samples were fractionated into six fractions. Gel pieces were destained with 50% ACN/50 mM Ammoniumcarbonat (ABC) and dehydrated with 100% ACN and dried via vacuum centrifugation. After rehydration in 10 ng/μL trypsin, 10% ACN/20 mM ABC, samples digestion was performed with trypsin o/n at 37 °C and 350 rpm. Protein digestion was stopped by adding 50% ACN/0.5% TFA. After an additional dehydration step, dried peptides were solved in 30 µL 2% ACN/0.1% TFA for LC/MS analysis.

The LC/MS analysis was performed using a Dionex Ultimate 3000 high-performance LC system and an LTQ Orbitrap Lumos mass spectrometer (Thermo Fisher Scientific, Waltham, MA, USA) in data-dependent acquisition (DDA) mode as described previously. Proteome data were analyzed using MaxQuant software (Version 1.6.50, https://www.maxquant.org/) and the human entries of the Uniprot database (https://www.uniprot.org/). Proteins were stated identified if the false discovery rate (FDR) on protein and peptide level was less than 0.01.

Network analyses were conducted using Ingenuity Pathway Analysis (release June 2018) using recommended parameters for the core analysis [43]. Networks were further manually edited.

### 4.2. Maintenance of Cell Lines

All cell lines were cultured at 37 °C and 5% CO_2_. The recombinant HLA class I negative K562 cell lines expressing HLA-F*01:0x (exon 1–6) were cultured in RPMI 1640 (Lonza, Basel, Switzerland) supplemented with 10% heat-inactivated fetal calf serum (FCS, Lonza, Basel, Switzerland), 2 mM L-glutamine (c. c. pro, Oberdorla, Germany), 100 U/mL penicillin and 100 µg/mL streptomycin (c. c. pro Oberdorla, Germany).

The human embryonal kidney cell line HEK239T (cell source ATCC, Manassas, VA, USA) was cultured in Dulbecco modified eagle medium (DMEM, Lonza, Basel, Switzerland) supplemented with 10% heat-inactivated FCS, 2 mM L-glutamine, 100 U/mL penicillin, 100 µg/mL streptomycin and 1 mg/mL Geneticin^®^ (Life Technologies, Carlsbad, CA, USA).

### 4.3. Cloning and Transduction of HLA-F Constructs

Construct encoding for HLA-F (exon 1–6) was generated from HEK293T cDNA via PCR. The sequence for HLA-F*01:01 was cloned into the lentiviral vector pRRL.PPT.SFFV.mcs.pre, as previously described [4]. To generate HLA-F*01:03 and HLA-F*01:04 encoding constructs, site-direct mutagenesis was used. Constructs for HLA-F*01:03 were generated by introducing a single point mutation at position c.814 T>C and at position c.212C>A for generating HLA-F*01:04. The constructs were verified through genomic sequencing.

Lentiviral particles were produced in *HEK293T* cells. *HEK293T* cells were transfected with the target plasmid for sHLA-F*01:0x (10 µg/5 × 10^6^ cells) along with the packaging and envelope vectors psPAX2 and pmD2G (each 5 µg/5 × 10^6^ cells). *K562* cells were stably transduced with lentiviral particles encoding for the different HLA-F variants. The expression of mHLA-F*01:0x molecules was confirmed by KIR3DS1-binding assay, as described in Section 4.4.

### 4.4. Detection of HLA-F on Transduced Cells via KIR3DS1-Binding Assay

For the detection of HLA-F on transduced cells, recombinant KIR3DS1-Fc chimeric protein (R & D Systems, Minneapolis, MN, USA) was used. 1 × 10^6^ K562/mF*01:0x cells were incubated in 100 µL Fc-block for 20 min at 4 °C. Afterward, the cells were incubated with KIR3DS1-Fc (25 µg/mL) for 40 min at 4 °C. To detect KIR3DS1-Fc chimeric protein on HLA-F, goat anti-human IgG Fc PE-conjugated antibody was utilized and incubated for 20 min at 4 °C. Flow cytometric acquisition was performed using a FACS Canto II flow cytometer (BD Biosciences, Heidelberg, Germany).

### 4.5. Peptide Binding Assay for HLA-F

For analysis of receptor HLA-F interaction, peptide-loading assays were performed. Initial bound peptides were removed from K562/HLA-F*01:0x transduced cells with citrate phosphate buffer. 2.5 × 10^5^ cells were incubated with 200 µM of peptide for 4 h at 37 °C and 5% CO_2_ in serum-free medium. To detect KIR3DS1, binding assays were performed as described in Section 4.4. Non-transduced *K562* cells served as a negative control (Figures S1–S3).

## Figures and Tables

**Table 1:** Strongest upregulated proteins in HIV-infected cells.

Protein Name	Gene Code	log_2_ Regulation	*p* Value
Carbonic anhydrase 1	CA1	7.01	<0.001
Integrin alpha-IIb	ITGA2B	5.59	<0.001
Hemoglobin subunit delta	HBD	4.83	<0.001
Hemoglobin subunit alpha	HBA1	4.67	<0.001
Hemoglobin subunit beta	HBB	4.67	<0.001
Flavin reductase (NADPH)	BLVRB	2.52	0.003
Myosin-9	MYH9	2.28	0.003
Myosin-14	MYH14	1.99	0.011
WD repeat-containing protein 61	WDR61	1.80	0.001
Pyridoxal kinase	PDXK	1.76	0.018

**Table 2:** Strongest downregulated proteins in HIV-infected cells.

Protein Name	Gene Code	log_2_ Regulation	*p* Value
Interferon-induced GTP-binding protein Mx1	MX1	−5.14	<0.001
Heat shock protein beta-1	HSPB1	−2.79	0.048
Ig gamma-1 chain C region	IGHG1	−2.55	0.026
Poly [ADP-ribose] polymerase 9	PARP9	−2.50	0.001
Myosin 1F	MYO1F	−2.00	0.014
Calmodulin Like 5	CALML5	−1.71	0.017
Retention In Endoplasmic Reticulum Sorting Receptor 1	RER1	−1.71	0.008
Mannose-P-Dolichol Utilization Defect 1	MPDU1	−1.69	0.001
Thymidine Phosphorylase	TYMP	−1.66	<0.001
Protein Tyrosine Phosphatase Non-Receptor Type 1	PTPN1	−1.58	0.020

## Supplementary Materials

The following are available online at https://www.mdpi.com/1422-0067/21/21/8012/s1.
