# Supplementary material for: The Loss of HLA-F/KIR3DS1 Ligation Is Mediated by Hemoglobin Peptides
Source: Int J Mol Sci. 2020 Oct 28;21(21):8012. doi: 10.3390/ijms21218012 (PMC7672607; doi:10.3390/ijms21218012)
Supplement: Supplementary file 1 [file ijms-21-08012-s001.pdf]

Supplementary material:

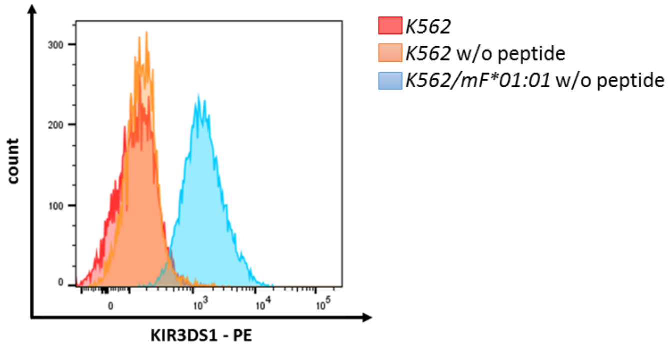

Figure S1: KIR3DS1 does not bind to *K562* cells. Red histogram shows KIR3DS1 binding to *K562* cells. Orange histogram shows KIR3DS1 binding to *K562* cells treated with citrate-phosphate buffer for peptide elution. Blue histogram shows KIR3DS1 binding to *K562/mF*01:01* cells treated with citrate-phosphate buffer for peptide elution.

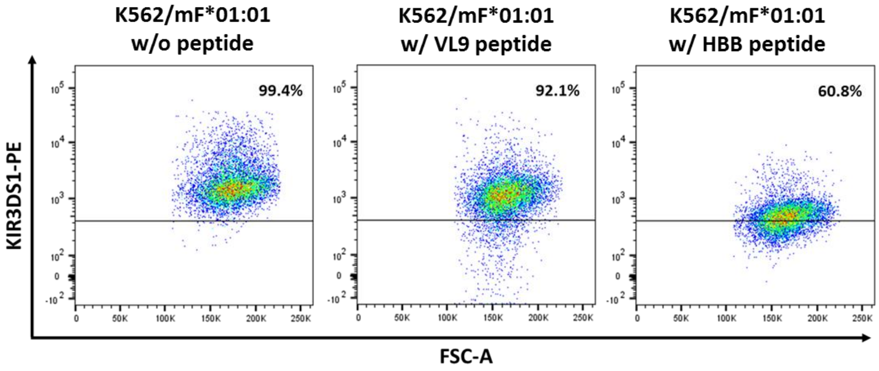

Figure S2: Exclusively the presentation of HLA-F restricted peptides reduced the binding of KIR3DS1. Incubation of HLA-F with peptide VL9 (VMAPRTLFL) does not reduce KIR3DS1 binding. K*562/mF*01:01* w/o peptide was used as positive control.

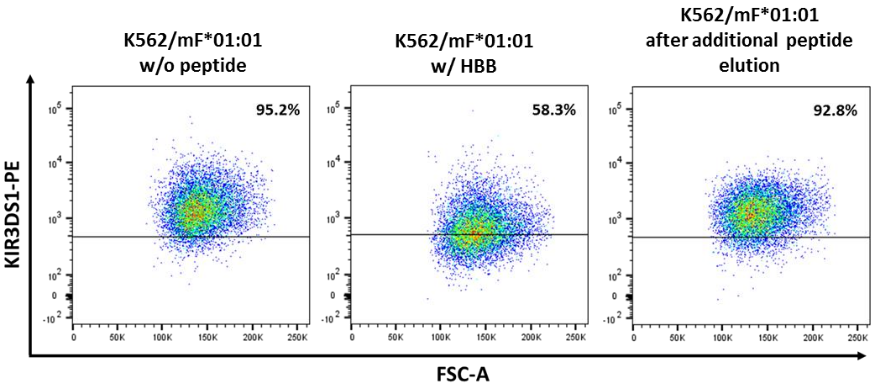

Figure S3: KIR3DS1 binding recurs after removal of the peptide. Incubation with HBB-peptide results in a reduction of KIR3DS1 binding. The removal of introduced peptide leads to the return of KIR3DS1 binding.
